# Demystifying the commercial determinants of health in antimicrobial resistance through complex system dynamics

**DOI:** 10.1093/heapro/daaf120

**Published:** 2025-08-12

**Authors:** Calum Smith, Jake Hitch, Lovro Savic

**Affiliations:** Nuffield Department of Population Health, University of Oxford, Oxford OX3 7LF, United Kingdom; Nuffield Department of Population Health, University of Oxford, Oxford OX3 7LF, United Kingdom; Nuffield Department of Population Health, University of Oxford, Oxford OX3 7LF, United Kingdom

**Keywords:** antimicrobial resistance, commercial determinants of health, complex system dynamics

## Abstract

Antimicrobial resistance (AMR) poses a significant threat to public health. The commercial determinants of health (CDoH) play a key role in shaping the development and growth of AMR. It is important to recognise these commercial factors and situate them within the complex system which describes the emergence and transmission of AMR, so that we can begin to evaluate their impact. There are several feedback loops in the AMR system that prevent a linear ‘cause and effect’ solution presenting itself to policymakers. These factors are all complexly interdependent and a solution requires recognition of this complexity. We take it for granted that one solution alone cannot tackle the issue of the growing threat of AMR. For this reason, we propose the use of complex system dynamics to visualize key interdependencies within the system. By building on existing systems maps of AMR, we propose to explore and highlight the relationships between the CDoH and AMR in order to demonstrate how commercial factors have consequences for and knock-on effects on other elements (and potential policy suggestions) within the system. In short, the proposed systems map is a tool that can be used to (i) represent some of the ways that commercial factors impact on AMR and (ii) visually simplify the complexity of the issue at hand. We provide a map that could act as a starting point to demonstrate our argument and act as a baseline to be developed going forward in collaboration with other research and non-research actors within the system.

Contribution to Health Promotion

- Antimicrobial resistance (AMR) is one of the biggest threats to global public health and development.
- The commercial determinants of health have a significant impact on the evolution, transmission, and burden of AMR.
- Commercial factors are both part of a broader range of drivers and consequences of AMR, and commercial factors often exacerbate other factors that are not traditionally seen as commercially driven.
- We provide a preliminary systems map to visually represent these relationships, and help actors within the system visualize the knock-on (and unforeseen) effects of their activities within the system.
- In order to understand the commercial determinants of AMR, we need to identify and examine commercial factors that impact AMR both directly and indirectly. This map can provide the starting point for further work in this area that examines AMR as an emergent property of a complex system in which CDoH plays a significant role.

## ANTIMICROBIAL RESISTANCE

Antimicrobial Resistance (AMR) occurs when bacteria, viruses, fungi, and parasites change over time and no longer respond to available antimicrobials used to prevent and treat infections (World Health Organization 2023). Although AMR is a natural evolutionary process, antibiotic use in human and animal populations ‘selects’ for resistance by inhibiting only those bacteria which have not developed a resistance mechanism, freeing up resources for bacteria which have. Antibiotic-resistant (or drug-resistant) bacteria can then spread within populations and disease (Review on Antimicrobial Resistance 2016). Methicillin-resistant Staphylococcus aureus is the most well-known example of a drug-resistant infection.

Drug-resistant infections are generally harder to treat, and resistance to available antibiotics is associated with additional health loss and healthcare costs. In 2021 there were an estimated 1.14 million deaths ‘attributable to’ bacterial AMR, meaning they would not have occurred if infections had been susceptible to available antibiotics (GBD 2021 Antimicrobial Resistance Collaborators 2024). By 2050, it is projected that more people will die from AMR each year than cancer (10 million vs 8.2 million) (Review on Antimicrobial Resistance 2016). Annual overall global healthcare expenditures could be $1.2 trillion higher in 2050 due to AMR (World Bank 2017). Through its effects on both human and animal populations, AMR is expected to have a significant overall economic impact. GDP is expected to be 1.1%–3.8% lower in 2050 due to AMR, depending on the scenario (World Bank 2017). Although current estimates have limitations, there is a general consensus that AMR is one of, if not the, most significant public health challenge of the twenty-first century.

To understand the potential contribution of commercial determinants of health (CDoH) to the overall burden of AMR, it is important to examine their role in the emergence of AMR and the transmission of AMR. In this article we focus on commercial actors and their interactions within the system through which AMR emerges and is transmitted. Although AMR can refer to viruses, fungi, and parasites, we focus on resistance of bacteria to antibiotics, due to the large, well-documented health and economic burden (Antimicrobial Resistance Collaborators 2022, GBD 2021 Antimicrobial Resistance Collaborators 2024).

## LINKS BETWEEN COMMERCIAL DETERMINANTS AND AMR

There are various definitions of CDoH (see de Lacy-Vawdon and Livingstone 2020). For this paper, we use the one outlined by Gilmore *et al*. (2023); the ‘systems, practices, and pathways through which commercial actors drive health and equity’. Naturally, this includes the systems, practices, and pathways through which commercial activities contribute to AMR. Development, manufacture and distribution of antibiotics, vaccines and infection prevention equipment, prescription, and use of antibiotics in hospitals and the community, and effluence of antibiotics into the natural environment are all contributing factors through which commercial actors and incentives shape AMR.

The WHO explicitly recognizes the link between intensive animal agriculture and AMR (World Health Organization 2017). Demand for animal products is linked with antibiotic use; higher demand (e.g. for meat) increases the use of antibiotics for growth promotion, and prevention and treatment of disease in animals. Particularly, intensive farming systems (with animals often living in intolerable conditions) adopted to meet food demand often rely on antimicrobials. Use of antimicrobials to reduce animal health risks and maximize productivity within animal farming can lead to the rapid growth and spread of resistant microorganisms (Ardakani *et al*. 2023), and the use of antibiotics that are clinically important for humans on animals can lead to resistance in animals and then spread to humans. This is in part a direct product of communities increasing disconnection from their food sources (Thomas *et al.* 2024).

One of the key practices and pathways through which commercial actors influence health outcomes linked to AMR is through direct participation in government decision-making around policy, i.e. lobbying. Madureira Lima and Galea (2018) point out that the US Department of Agriculture receives financial contributions from agribusiness, and that antibiotic use for meat growth is voluntarily regulated by those with vested interests (e.g. meat and pharmaceutical industries). Specifically, in this context, vested interests (such as the US Department of Agriculture historically having staff who have a background in meat production) can lead to acceptance of the model of self-regulation by meat and pharmaceutical businesses ‘instead’ of potentially more positively impactful statutory regulation by governments (Madureira Lima and Galea 2018). If correct, this would be a clear case of commercial factors taking precedence over the most effective public health measures.

Similarly, Donovan (2015) argues that the Food and Drug Administration is currently under-regulating its food, given that modern meat production allows pathogens to spread with ease. Both ‘revolving doors’ and lobbying allow for those that financially benefit from continuation of the status quo to prioritize treatment over prevention, instead of higher levels of regulation that may positively impact on public health goals (potentially) at the expense of financial interests of, e.g. those involved in meat production.

We have focused on meat production as a key commercial actor within this space, however, this is not to imply that this is the main (or only actor). Some other key areas where CDoH impact on AMR include:

- Antimicrobial development: Ensuring a robust antimicrobial pipeline is a key part of the solution to AMR (World Health Organization 2022). However, due to public health concerns, new antibiotics will generally be conserved to sustain their effectiveness and act as last-resort treatment. Stewardship makes antimicrobial development a relatively unattractive area of research and development for the commercial pharmaceutical industry (Simpkin *et al.* 2017).
- Infection prevention and control in healthcare settings: Another key part of the solution to AMR is reducing the transmission of infections, whether they are susceptible to antibiotics or not. The burden of healthcare-associated drug-resistant infections is high (Suetens *et al.* 2018). This makes infection prevention and control measures, including hygiene and the use of personal protective equipment, particularly important, and commercial actors are involved in this supply chain.
- Vaccine development: It is increasingly recognized that vaccines have a potentially large role to play in addressing AMR (Micoli *et al.* 2021). Vaccines against bacterial pathogens will reduce the transmission of drug-resistant infections and vaccines against viral pathogens will help reduce mis-prescribing of antibiotics, particularly in primary care, and hence reduce selection pressure on bacteria. The pharmaceutical industry is generally responsible for most vaccine development and manufacturing.
- Environmental contamination: Antibiotics can enter the environment through inappropriate disposal and handling of unused drugs, aquaculture and plant production, and waste from antibiotic production (Larsson and Flach 2022). There are commercial actors involved in all three of these areas, including private companies contracted by healthcare providers, animal food producers, and the pharmaceutical industry. As Thomas *et al.* note, pollution of water, soil, and air are linked to commercial factors, posing a significant harm to both human and planetary health (Thomas *et al.* 2024)

Policymakers and others identifying solutions to threats posed by AMR need be cognizant of commercial impacts on AMR. However, we recognize that these systems and pathways can be complex and often opaque. Therefore, we propose the use of complex systems dynamics methodology to help visualize and aid comprehension of these relationships.

## METHODOLOGY

### System dynamics

System dynamics (SD) is a methodology that is used as a way of dealing with complex (also known as ‘wicked’) problems (Sterman 2001). Understanding complexity, predicting behaviours of systems and devising modifications within this complex whole are all key elements of systems thinking (Arnold and Wade 2015). When problems are best examined non-linearly by taking into account the interactivity between multiple different parts of the synergistic whole, it may be useful to deploy SD (Arnold and Wade 2015). Pruyt (2013) describes SD as a way of attempting to ‘describe, model, simulate, and analyse dynamically complex issues*’* (Pruyt 2013). It is a way, for Pruyt, of identifying the processes, information, and organizational boundaries that occur in such a way as to synergistically create a ‘complex’ problem. SD, then, is a way of identifying and untangling the relationship between different elements of a problem, and trying to find a way towards a solution by looking at the interrelationships between different components (Sterman 2001).

First, a scoping review was conducted in April 2024. Papers not older than 10 years were selected (2014–24). From this literature, potential nodes within the system were identified, and then these elements were connected using positive and negative feedback loops. Next, we analysed other existing maps to identify any nodes that were missing from our initial map. Finally, using the literature we identified which nodes (and loops) could be classified as ‘CDoH’, and colour-coded them accordingly.

### Existing maps

Three other causal loop diagrams express how AMR is an emergent property of a complex system (Department of Health 2014, Matthiessen *et al*. 2022, World Health Organization 2024). Each references commercial driven factors, such as human food (Department of Health 2014), intensified animal production (Matthiessen *et al*. 2022), and manufacturing and production of pharmaceuticals (World Health Organization 2024). Our map builds on these by outlining how elements such as lobbying, advertising, regulation of food environments, etc. impact on AMR; using CDoH language to reveal how actions to tackle the CDoH explicitly could impact on the emergence of AMR. For example, within our map, the nodes indicate that with increased lobbying, there is likely to be decreased regulation of the food environment (these move in opposite directions as indicated by a dotted line). With less regulation of the food environment, there is increased ‘Advertising’, which in turn increases ‘Demand for animal products in diet’, which leads to increased ‘Antibiotic use in animals’, leading to increased ‘AMR in Animals’ and ‘AMR in Natural Environment’.

### The map

**Figure 1.**
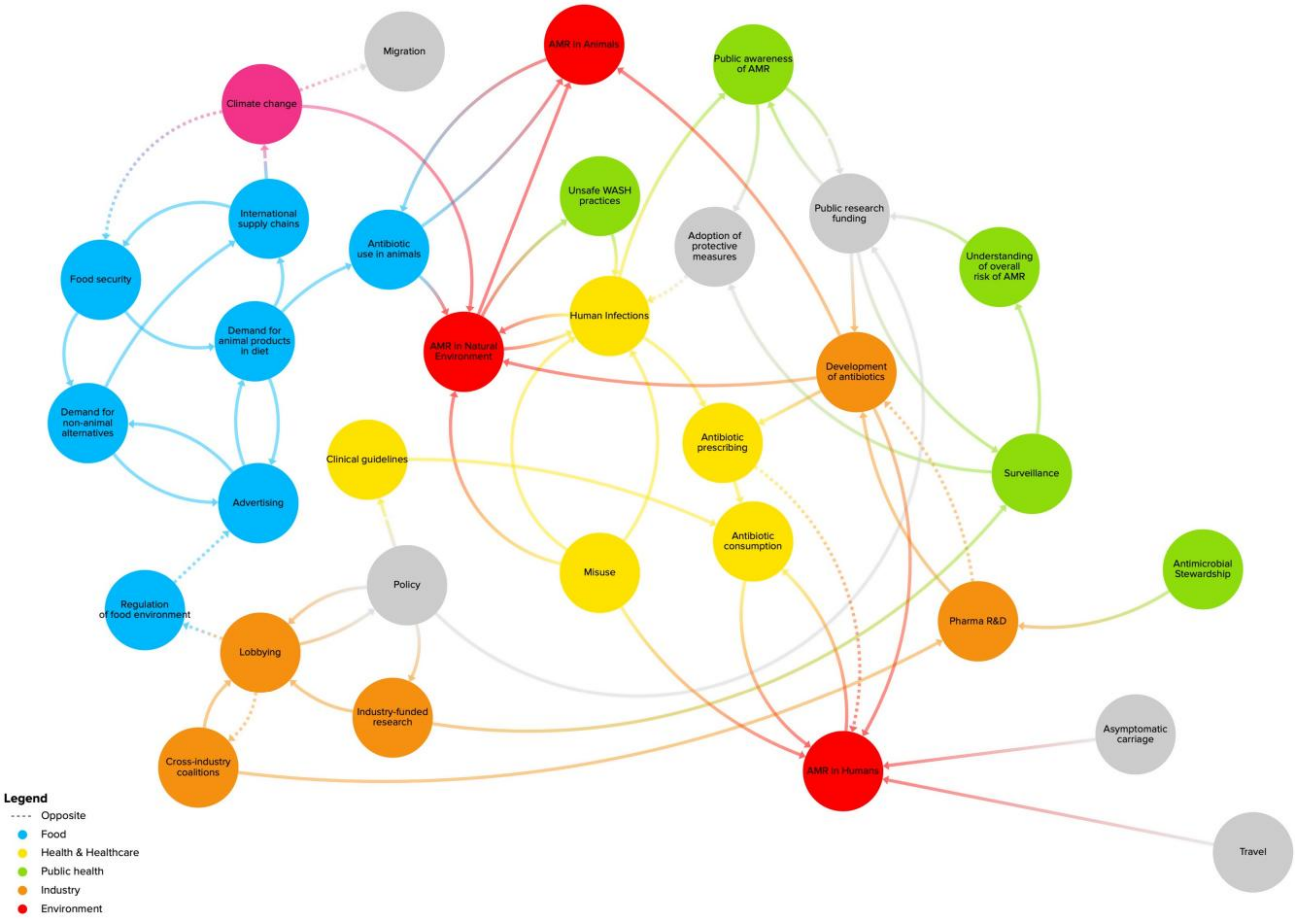
AMR systems map (with CDoH elements appearing towards the left of the map).

In our map (Fig. 1), nodes (Table 1) represent variable factors in the AMR system and lines represent causal relationships and loops (Table 2) which may be bidirectional. Naturally, some elements of the system are interconnected to, but not necessarily directly identified as, CDoH elements. What we feature here is by no means an exhaustive map representing all elements and loops within the AMR system. Rather, it is a starting point that begins to illuminate how CDoH play a key role within AMR and identifies how CDoH are related to elements of the AMR system that are not necessarily perceived as being obviously related to CDoH.

**Table 1.** Explanation of nodes in the system.

Node	Explanation
Food security	Population’s reliable access to sufficient amount of food
International supply chains	Supply chains that transverse international borders
Antibiotic use in animals	Use of antibiotics in animals for medical or agricultural (including food production) purposes
Demand for animal products in diet	Population demand for animal-based food and drink products, e.g. meat and dairy products
Advertising	By which, e.g. food companies can encourage consumers to desire their product
Regulation of food environment	By, e.g. governments who can legislate around content of food products, advertising regulation etc.
Lobbying	Act of lawfully impacting regulation/policy
Industry funded research	Research funded by companies (sometimes a source of conflicts of interest)
Cross industry coalitions	Coalitions of private companies set up to facilitate collaboration on AMR, e.g. AMR Industry Alliance
Policy	Principle of action proposed by government
Clinical guidelines	Recommendations for medical professionals on how to diagnose and treat medical conditions, e.g. suspected bacterial infections
Antibiotic consumption	Human consumption of antibiotics, whether prescribed or not, for the purpose of disease prevention and treatment
Antibiotic prescribing	Prescription of antibiotics by doctors and other medical professionals. Antibiotic prescribing is not necessarily equal to antibiotic consumption due to misuse and non-prescribed antibiotic use
Misuse	Combination of inappropriate antibiotic prescribing, e.g. for viral infections, and inappropriate antibiotic consumption e.g. not completing full course
Human infections	Viral and bacterial infections in humans (stock at any given time)
Climate change	Global warming and more volatile weather patterns
Antibiotic traces in nature	Presence of antibiotics in the natural environment e.g. from runoff from farms, and effluence from hospitals and drug manufacturing sites
Unsafe WASH practices	Water, sanitation and hygiene practices that do not best promote health
Migration	Movement of peoples from one region to another
Public awareness of AMR	The general public’s understanding and perception of the risk posed by AMR, and the necessity to act in response
Understanding of overall risk of AMR	Perception (amongst public and policymakers) of actual threat posed at any given time to population health by AMR
Surveillance	Collection, analysis and communication of AMR data, e.g. prevalence, transmission, and resistance to antibiotics
Pharma R&D	Research and development into the discovery and development of new antibiotics conducted by and funded by the pharmaceutical industry

**Table 2.** Explanation of selected loops in the system*.

Loop	Explanation
Antibiotic consumption > AMR in humans	Human antibiotic consumption contributes AMR in humans through selection for drug-resistant bacteria. Greater prevalence of drug-resistant infections is likely to result in increased antibiotic consumption through the requirement for additional courses of antibiotics and longer durations of infections resulting in more transmission. Antibiotic consumption is affected by commercial factors such as development and distribution of antibiotics, and physician prescribing which may be partly commercially driven depending on the country
Antimicrobial stewardship > antibiotic prescribing > development of new antibiotics	Antimicrobial stewardship partly involves conserving newer, last-resort antibiotics. While it is sensible to conserve the effectiveness of new antibiotics, this effectively limits the expected market for novel antibiotics, negatively impacting incentives for pharmaceutical and biotechnology companies to invest in R&D in this therapeutic area. Given that antibiotics are likely to remain the main treatment for bacterial infections for some time, a dry antibiotic pipeline has negative implications for the prevalence of drug-resistant infections
Food security > demand for animal products in diet > international supply chains	Increased food security increases demand for (more and higher quality) animal products in diet, which leads to the propagation of international supply chains to cater for this need. We can see that (through the demand for animal products in diet) this seemingly self-contained reinforcing feedback loop has its own impact on AMR in animals.
Demand for animal products in diet > advertising	This two-node reinforcing feedback loop (more advertising leads to higher demand, higher demand leads to more advertising (as it is perceived to be effective)) is within the food system. We can see that it has knock-on impacts on a range of other elements within the system (we can, e.g. trace demand for animal products in diet to antibiotic use in animals, to AMR in the natural environment (through waste), and into human infections, and so on
Antibiotic use in animals > AMR in animals	This two-node reinforcing feedback loop (whereby more antibiotic use in animals leads to more AMR in animals, requiring the use of more antibiotic use in animals) is not CDoH-driven when we look at the self-contained loop, but we can see that it is clearly driven by demand for animal products in diet, which in turn is driven by advertising, and so on

*We do not have space to elucidate on all of the loops and elements of feedback within the system. Instead, we have chosen to focus on a select few elements that we perceive to be central to AMR and/or the relationship between CDoH and AMR.

## DISCUSSION

Though the label CDoH has not often been applied to AMR, there are many systems, pathways, and practices through which commercially driven actions contribute to the health burden of AMR. A systems approach makes many of these linkages explicit. Importantly, AMR has often been described as an example of the tragedy of the commons or an externality problem (Hollis and Maybarduk 2015, Giubilini 2019), and a systems approach can be a useful way to visualize and track the propagation of these externalities through the system.

While the main theoretical benefit of using SD rests on the ability to explicate complexity and a range of different commercial processes and pathways that contribute to or otherwise exacerbate AMR, perhaps the most obvious example involves a key set of relationships between CDoH and AMR that are found in animal-based food industries. For example, without changes to policy or behaviour, global meat consumption is expected to increase 60%–70% from 2018 levels by 2050 (Springmann *et al*. 2018). This is in part due to income growth in the global south and is likely to increase demand for antibiotics for disease management and growth promotion. Livestock and fish are a significant reservoir for AMR and drug-resistant bacteria can spread from animals to humans through food, and indirectly through the natural environment (Ibekwe *et al*. 2023). This makes agriculture a key industry in the AMR system.

Advertising to promote meat-based products can lead to larger livestock populations and, all else being equal, more animal antibiotic use. This can then spill over into the environment and human populations. These are negative spillovers. As Africa grows richer, global demand for animal-based food products is likely to increase, leading to larger livestock populations. Simultaneously, demand continues to grow in the global north, both for animal products, animal product alternatives, and non-animal products. Through complex pathways (not all of them, we admit, captured in our map), many of these products rely (through an international supply chain) on the continued use of antibiotics in animal and plant agriculture.

This map (and the linkage of AMR and CDoH) has particular relevance when discussing theoretical increased antibiotic use in low- and middle-income countries (LMICs), where the global burden of AMR is concentrated. If recommendations are for reduction or cessation of use of antibiotics for growth promotion in animals and fish, this may be acceptable in high income countries (HIC) but becomes problematic when discussing food access and global equity qua food production in LMICs. The ethical considerations surrounding this are beyond the scope of this paper, but are central to the debates around the links between CDoH and AMR going forward. As Thomas *et al.* state, a key aim for the CDoH moving forward ought recognize and address how health harms are disproportionately concentrated in LMICs (Thomas *et al*. 2024).

Maps of this sort have utility for policymakers and other agents within the system. With a preliminary model such as this, agents within the system will be able to visualize and better understand their interlinkages to other ‘nodes’ within the system. Inspired by Meadows’ seminal ‘world modelling’ work in ‘Limits to Growth’ (Meadows *et al*. 2004), the model will aim to demonstrate how policy change/regulation of activities in one sector have knock-on impacts within other areas of the system. In creating a map visualizing how AMR can occur as an emergent property from a complex system that includes CDoH elements, we hope to illuminate ways that AMR-related CDoH ought to be addressed, and demonstrate to policymakers that when it comes to AMR, CDoH cannot be ignored.

This map also has the potential to demonstrate something which is gaining substantial interest within the field of CDoH. Namely, bridging the gap between CDoH and both communicable and non-communicable diseases (NCDs). AMR renders infections harder to treat. NCDs weaken immune systems and lead to more contact with healthcare settings where they may contract drug-resistant infections. AMR directly impacts the treatment of infections and infectious syndromes, but many NCDs are key risk factors for infection, and therefore are an important consideration.

### Limitations

By the nature of trying to capture the system and demonstrate how CDoH factors link with non-CDoH factors, our map has required a significant amount of collapsing of non-homogenous activities into single nodes. For example, we have included one node for ‘Advertising’, which is a complex and heterogeneous activity, one for ‘Policy’, which itself involves many actors and large time frames, and ‘Demand for animal products in diet’, which as a single node does not fully demonstrate the complexities involved in this demand, such as iniquitous access to healthy diets. These are but a few illustrative examples of oversimplifications and homogenizations that we have included that do not necessarily replicate the complexity in the real world. We have done this for explanatory simplicity, yet acknowledge that a truer, more useful map would begin to unpack these (and other) nodes fuller to highlight more emergent feedback loops.

Additionally, given the complexity of the AMR system, there are many (endogenous and exogenous) variables that we have not included in this analysis. A tightrope must be walked between clarity and comprehensiveness. We have tried to tread this line carefully, but acknowledge that in doing so there are pathways through which commercial actors impact on AMR that are missing. For example, historically unjust practices by actors in the global north have led to what Farmer calls ‘healthcare deserts’ in the global south that have had an impact on morbidity and antibiotic use today. This is an ever-present variable left out of the current analysis, though this does not indicate that we do not view it as important.

Finally, the explanatory and practical benefits of our approach could be augmented significantly by inputting figures into the causal loop diagram (CLD) and ‘running’ the map through software. This could help demonstrate the growth of loops and knock-on effects on other areas of the system. Future research could, for example, aim to create a ‘stock and flow’ diagram of the link between CDoH and AMR. At present our map is static, presenting variables without inputting quantitative metrics that could help demonstrate change over time, though this would, we believe, be a useful direction for future research.

## CONCLUSION

CDoH are important in AMR. Complex SD can play a valuable role within CDoH efforts to examine the systems and pathways through which commercial activities impact upon health. Through these feedback loops, interconnections, and separate themes, we are able to recognize AMR as an emergent property of a complex system within which CDoH plays a relevant role. The value of SD lies in the ability to highlight the complexity and non-linearity of the causal mechanisms that underlie such a significant public health threat.

Further public health research is required to explore the relationship between CDoH and AMR. Specific examples and efforts to highlight the positive impact that commercial activities have on healthcare and development of new antimicrobials (and tackling infections) are invaluable, alongside potentially problematic activities that exacerbate the threat posed by AMR. We hope that this systems map provides a starting point that helps explain and demystify the relationship between CDoH and AMR, and between CDoH and non-CDoH complex drivers of AMR.

## Data Availability

The authors confirm that all data generated or analysed during this study are included in this published article. Furthermore, primary and secondary sources and data supporting the findings of this study were all available at the time of submission.
